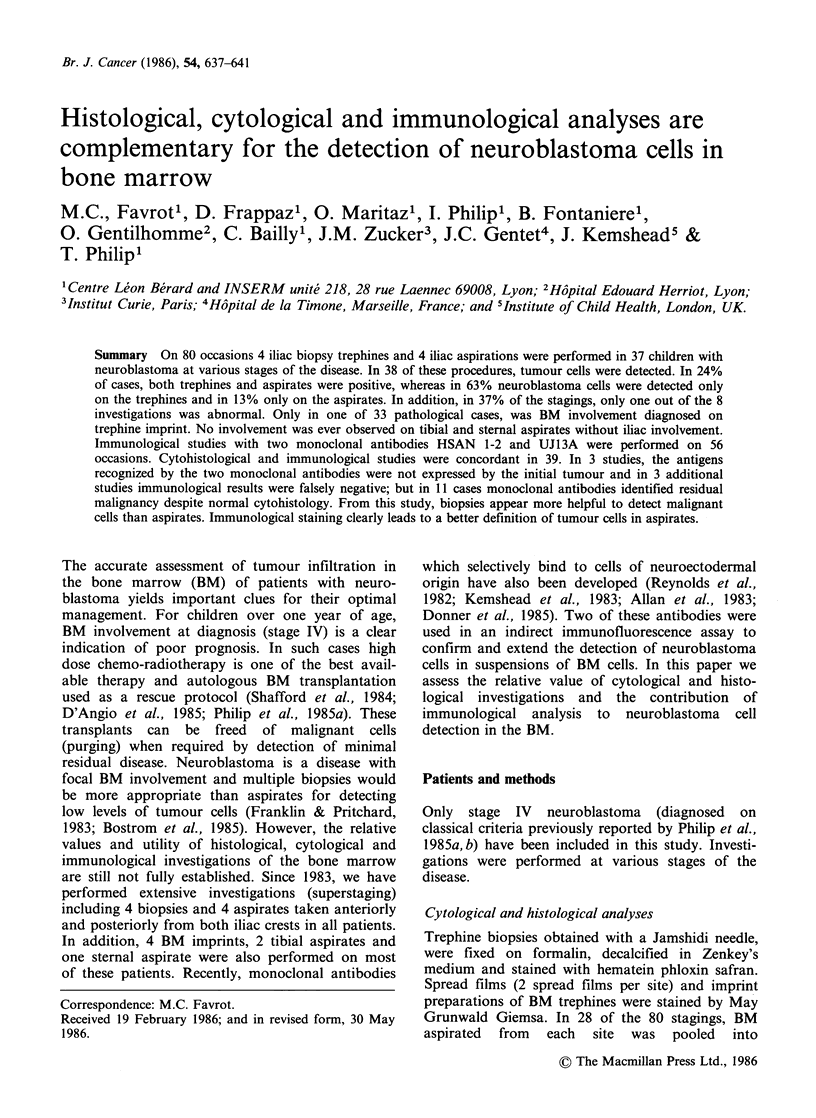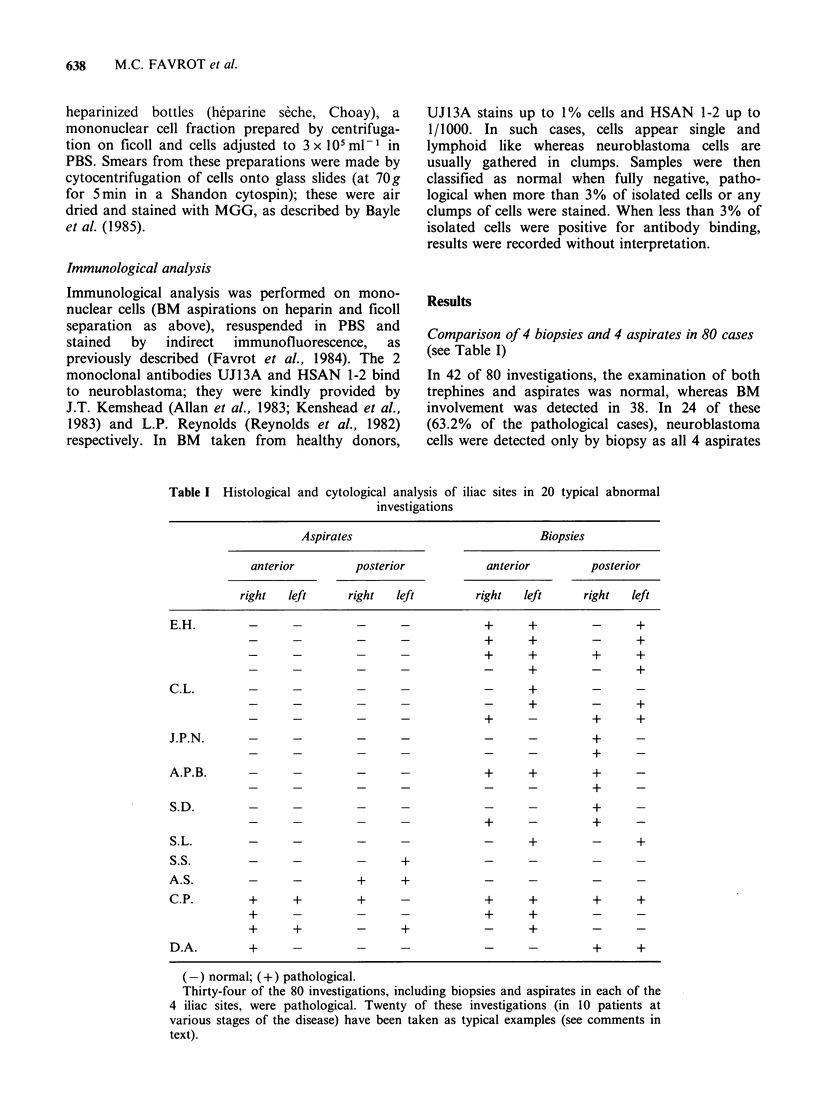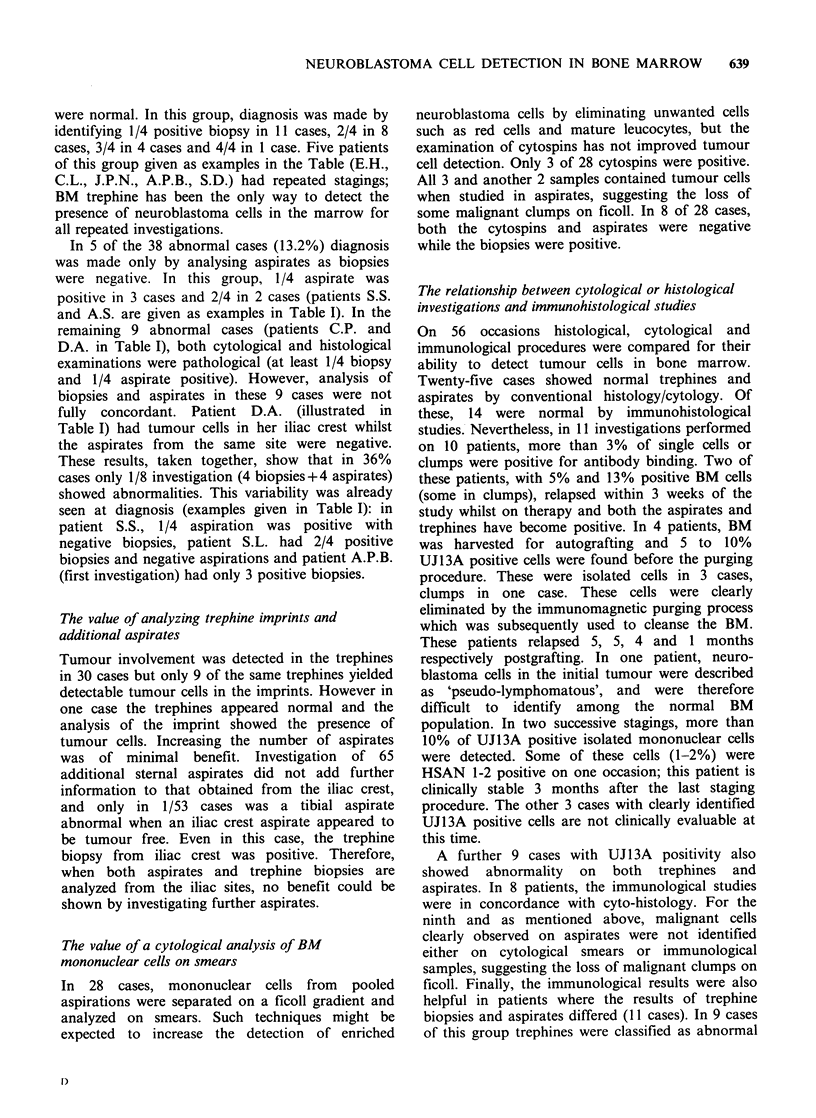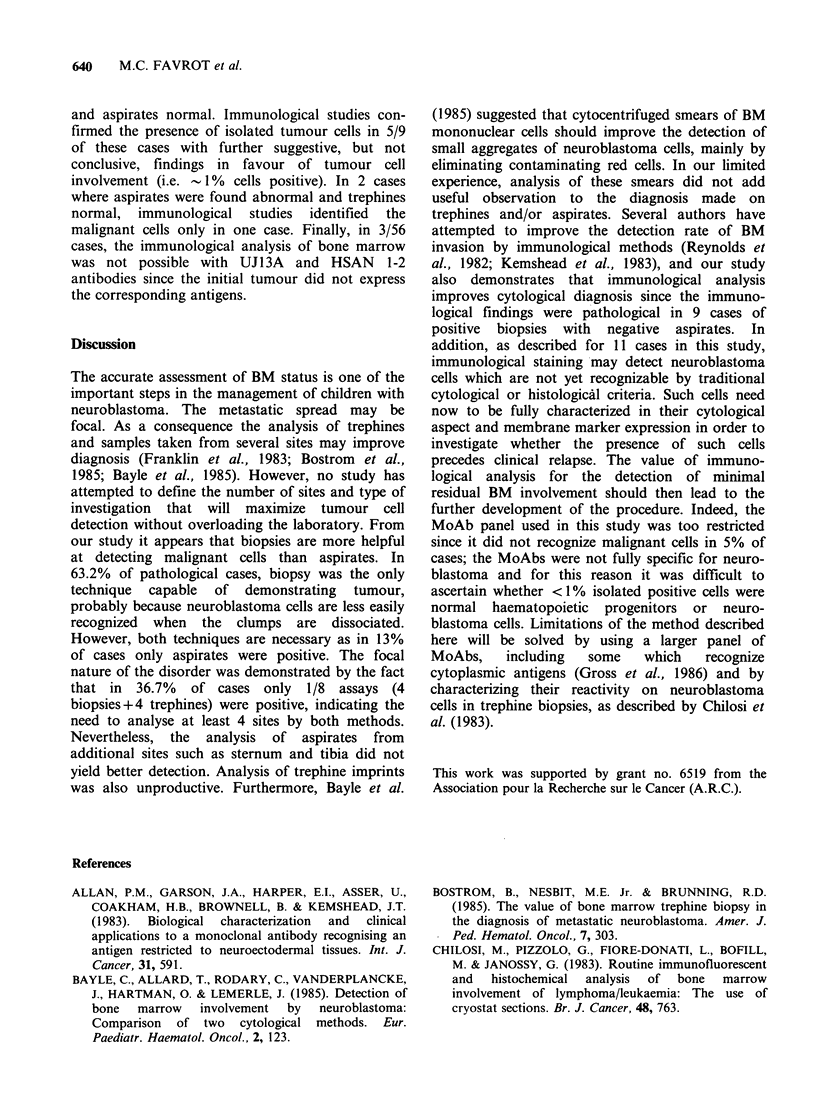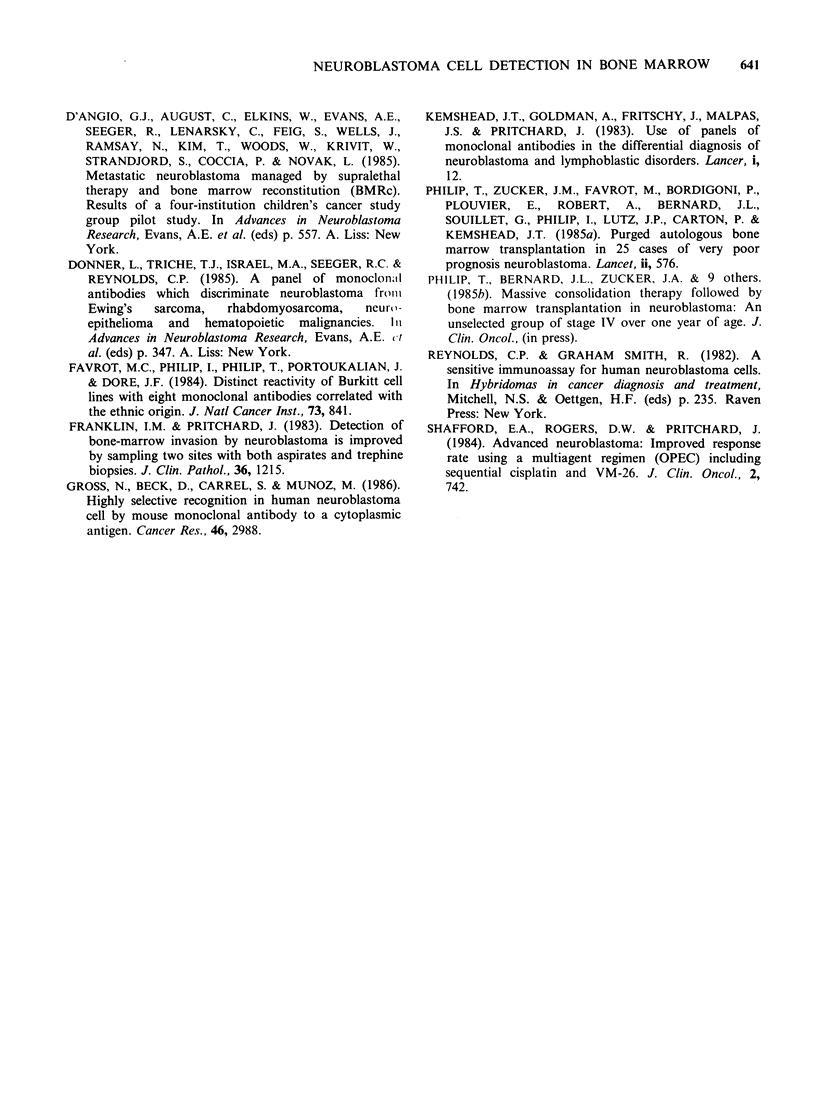# Histological, cytological and immunological analyses are complementary for the detection of neuroblastoma cells in bone marrow.

**DOI:** 10.1038/bjc.1986.220

**Published:** 1986-10

**Authors:** M. C. Favrot, D. Frappaz, O. Maritaz, I. Philip, B. Fontaniere, O. Gentilhomme, C. Bailly, J. M. Zucker, J. C. Gentet, J. Kemshead

## Abstract

On 80 occasions 4 iliac biopsy trephines and 4 iliac aspirations were performed in 37 children with neuroblastoma at various stages of the disease. In 38 of these procedures, tumour cells were detected. In 24% of cases, both trephines and aspirates were positive, whereas in 63% neuroblastoma cells were detected only on the trephines and in 13% only on the aspirates. In addition, in 37% of the stagings, only one out of the 8 investigations was abnormal. Only in one of 33 pathological cases, was BM involvement diagnosed on trephine imprint. No involvement was ever observed on tibial and sternal aspirates without iliac involvement. Immunological studies with two monoclonal antibodies HSAN 1-2 and UJ13A were performed on 56 occasions. Cytohistological and immunological studies were concordant in 39. In 3 studies, the antigens recognized by the two monoclonal antibodies were not expressed by the initial tumour and in 3 additional studies immunological results were falsely negative; but in 11 cases monoclonal antibodies identified residual malignancy despite normal cytohistology. From this study, biopsies appear more helpful to detect malignant cells than aspirates. Immunological staining clearly leads to a better definition of tumour cells in aspirates.


Br. J. Cancer (1986), 54, 637–641

### Histological, cytological and immunological analyses are complementary for the detection of neuroblastoma cells in bone marrow

M.C., Favrot[1], D. Frappaz[1], O. Maritaz[1], I. Philip[1], B. Fontaniere[1], O. Gentilhomme[2], C. Bailly[1], J.M. Zucker[3], J.C. Gentet[4], J. Kemshead[5] & T. Philip[1]

[1]*Centre Léon Bérard and INSERM unité 218, 28 rue Laennec 69008, Lyon;* [2]*Hôpital Edouard Herriot, Lyon;* [3]*Institut Curie, Paris;* [4]*Hôpital de la Timone, Marseille, France; and* [5]*Institute of Child Health, London, UK.*

**Summary** On 80 occasions 4 iliac biopsy trephines and 4 iliac aspirations were performed in 37 children with neuroblastoma at various stages of the disease. In 38 of these procedures, tumour cells were detected. In 24% of cases, both trephines and aspirates were positive, whereas in 63% neuroblastoma cells were detected only on the trephines and in 13% only on the aspirates. In addition, in 37% of the stagings, only one out of the 8 investigations was abnormal. Only in one of 33 pathological cases, was BM involvement diagnosed on trephine imprint. No involvement was ever observed on tibial and sternal aspirates without iliac involvement. Immunological studies with two monoclonal antibodies HSAN 1-2 and UJ13A were performed on 56 occasions. Cytohistological and immunological studies were concordant in 39. In 3 studies, the antigens recognized by the two monoclonal antibodies were not expressed by the initial tumour and in 3 additional studies immunological results were falsely negative; but in 11 cases monoclonal antibodies identified residual malignancy despite normal cytohistology. From this study, biopsies appear more helpful to detect malignant cells than aspirates. Immunological staining clearly leads to a better definition of tumour cells in aspirates.

The accurate assessment of tumour infiltration in the bone marrow (BM) of patients with neuroblastoma yields important clues for their optimal management. For children over one year of age, BM involvement at diagnosis (stage IV) is a clear indication of poor prognosis. In such cases high dose chemo-radiotherapy is one of the best available therapy and autologous BM transplantation used as a rescue protocol (Shafford *et al.*, 1984; D'Angio *et al.*, 1985; Philip *et al.*, 1985*a*). These transplants can be freed of malignant cells (purging) when required by detection of minimal residual disease. Neuroblastoma is a disease with focal BM involvement and multiple biopsies would be more appropriate than aspirates for detecting low levels of tumour cells (Franklin & Pritchard, 1983; Bostrom *et al.*, 1985). However, the relative values and utility of histological, cytological and immunological investigations of the bone marrow are still not fully established. Since 1983, we have performed extensive investigations (superstaging) including 4 biopsies and 4 aspirates taken anteriorly and posteriorly from both iliac crests in all patients. In addition, 4 BM imprints, 2 tibial aspirates and one sternal aspirate were also performed on most of these patients. Recently, monoclonal antibodies which selectively bind to cells of neuroectodermal origin have also been developed (Reynolds *et al.*, 1982; Kemshead *et al.*, 1983; Allan *et al.*, 1983; Donner *et al.*, 1985). Two of these antibodies were used in an indirect immunofluorescence assay to confirm and extend the detection of neuroblastoma cells in suspensions of BM cells. In this paper we assess the relative value of cytological and histological investigations and the contribution of immunological analysis to neuroblastoma cell detection in the BM.

Correspondence: M.C. Favrot.
Received 19 February 1986; and in revised form, 30 May 1986.

### Patients and methods

Only stage IV neuroblastoma (diagnosed on classical criteria previously reported by Philip *et al.*, 1985*a,b*) have been included in this study. Investigations were performed at various stages of the disease.

### *Cytological and histological analyses*

Trephine biopsies obtained with a Jamshidi needle, were fixed on formalin, decalcified in Zenkey's medium and stained with hematein phloxin safran. Spread films (2 spread films per site) and imprint preparations of BM trephines were stained by May Grunwald Giemsa. In 28 of the 80 stagings, BM aspirated from each site was pooled into

© The Macmillan Press Ltd., 1986

heparinized bottles (héparine sèche, Choay), a mononuclear cell fraction prepared by centrifugation on ficoll and cells adjusted to $3 \times 10^5 ml^{-1}$ in PBS. Smears from these preparations were made by cytocentrifugation of cells onto glass slides (at 70 *g* for 5 min in a Shandon cytospin); these were air dried and stained with MGG, as described by Bayle *et al.* (1985).

*Immunological analysis*

Immunological analysis was performed on mononuclear cells (BM aspirations on heparin and ficoll separation as above), resuspended in PBS and stained by indirect immunofluorescence, as previously described (Favrot *et al.*, 1984). The 2 monoclonal antibodies UJ13A and HSAN 1-2 bind to neuroblastoma; they were kindly provided by J.T. Kemshead (Allan *et al.*, 1983; Kenshead *et al.*, 1983) and L.P. Reynolds (Reynolds *et al.*, 1982) respectively. In BM taken from healthy donors, UJ13A stains up to 1% cells and HSAN 1-2 up to 1/1000. In such cases, cells appear single and lymphoid like whereas neuroblastoma cells are usually gathered in clumps. Samples were then classified as normal when fully negative, pathological when more than 3% of isolated cells or any clumps of cells were stained. When less than 3% of isolated cells were positive for antibody binding, results were recorded without interpretation.

### Results

*Comparison of 4 biopsies and 4 aspirates in 80 cases* (see Table I)

In 42 of 80 investigations, the examination of both trephines and aspirates was normal, whereas BM involvement was detected in 38. In 24 of these (63.2% of the pathological cases), neuroblastoma cells were detected only by biopsy as all 4 aspirates

**Table I** Histological and cytological analysis of iliac sites in 20 typical abnormal investigations

| | *Aspirates* | | | | *Biopsies* | | | |
|---|---|---|---|---|---|---|---|---|
| | *anterior* | | *posterior* | | *anterior* | | *posterior* | |
| | *right* | *left* | *right* | *left* | *right* | *left* | *right* | *left* |
| E.H. | − | − | − | − | + | + | − | + |
| | − | − | − | − | + | + | − | + |
| | − | − | − | − | + | + | + | + |
| | − | − | − | − | − | + | − | + |
| C.L. | − | − | − | − | − | + | − | − |
| | − | − | − | − | − | + | − | + |
| | − | − | − | − | + | − | + | + |
| J.P.N. | − | − | − | − | − | − | + | − |
| | − | − | − | − | − | − | + | − |
| A.P.B. | − | − | − | − | + | + | + | − |
| | − | − | − | − | − | − | + | − |
| S.D. | − | − | − | − | − | − | + | − |
| | − | − | − | − | + | − | + | − |
| S.L. | − | − | − | − | − | + | − | + |
| S.S. | − | − | − | + | − | − | − | − |
| A.S. | − | − | + | + | − | − | − | − |
| C.P. | + | + | + | − | + | + | + | + |
| | + | − | − | − | + | + | − | − |
| | + | + | − | + | − | + | − | − |
| D.A. | + | − | − | − | − | − | + | + |

(−) normal; (+) pathological.

Thirty-four of the 80 investigations, including biopsies and aspirates in each of the 4 iliac sites, were pathological. Twenty of these investigations (in 10 patients at various stages of the disease) have been taken as typical examples (see comments in text).

were normal. In this group, diagnosis was made by identifying 1/4 positive biopsy in 11 cases, 2/4 in 8 cases, 3/4 in 4 cases and 4/4 in 1 case. Five patients of this group given as examples in the Table (E.H., C.L., J.P.N., A.P.B., S.D.) had repeated stagings; BM trephine has been the only way to detect the presence of neuroblastoma cells in the marrow for all repeated investigations.

In 5 of the 38 abnormal cases (13.2%) diagnosis was made only by analysing aspirates as biopsies were negative. In this group, 1/4 aspirate was positive in 3 cases and 2/4 in 2 cases (patients S.S. and A.S. are given as examples in Table I). In the remaining 9 abnormal cases (patients C.P. and D.A. in Table I), both cytological and histological examinations were pathological (at least 1/4 biopsy and 1/4 aspirate positive). However, analysis of biopsies and aspirates in these 9 cases were not fully concordant. Patient D.A. (illustrated in Table I) had tumour cells in her iliac crest whilst the aspirates from the same site were negative. These results, taken together, show that in 36% cases only 1/8 investigation (4 biopsies + 4 aspirates) showed abnormalities. This variability was already seen at diagnosis (examples given in Table I): in patient S.S., 1/4 aspiration was positive with negative biopsies, patient S.L. had 2/4 positive biopsies and negative aspirations and patient A.P.B. (first investigation) had only 3 positive biopsies.

*The value of analyzing trephine imprints and additional aspirates*

Tumour involvement was detected in the trephines in 30 cases but only 9 of the same trephines yielded detectable tumour cells in the imprints. However in one case the trephines appeared normal and the analysis of the imprint showed the presence of tumour cells. Increasing the number of aspirates was of minimal benefit. Investigation of 65 additional sternal aspirates did not add further information to that obtained from the iliac crest, and only in 1/53 cases was a tibial aspirate abnormal when an iliac crest aspirate appeared to be tumour free. Even in this case, the trephine biopsy from iliac crest was positive. Therefore, when both aspirates and trephine biopsies are analyzed from the iliac sites, no benefit could be shown by investigating further aspirates.

*The value of a cytological analysis of BM mononuclear cells on smears*

In 28 cases, mononuclear cells from pooled aspirations were separated on a ficoll gradient and analyzed on smears. Such techniques might be expected to increase the detection of enriched neuroblastoma cells by eliminating unwanted cells such as red cells and mature leucocytes, but the examination of cytospins has not improved tumour cell detection. Only 3 of 28 cytospins were positive. All 3 and another 2 samples contained tumour cells when studied in aspirates, suggesting the loss of some malignant clumps on ficoll. In 8 of 28 cases, both the cytospins and aspirates were negative while the biopsies were positive.

*The relationship between cytological or histological investigations and immunohistological studies*

On 56 occasions histological, cytological and immunological procedures were compared for their ability to detect tumour cells in bone marrow. Twenty-five cases showed normal trephines and aspirates by conventional histology/cytology. Of these, 14 were normal by immunohistological studies. Nevertheless, in 11 investigations performed on 10 patients, more than 3% of single cells or clumps were positive for antibody binding. Two of these patients, with 5% and 13% positive BM cells (some in clumps), relapsed within 3 weeks of the study whilst on therapy and both the aspirates and trephines have become positive. In 4 patients, BM was harvested for autografting and 5 to 10% UJ13A positive cells were found before the purging procedure. These were isolated cells in 3 cases, clumps in one case. These cells were clearly eliminated by the immunomagnetic purging process which was subsequently used to cleanse the BM. These patients relapsed 5, 5, 4 and 1 months respectively postgrafting. In one patient, neuroblastoma cells in the initial tumour were described as 'pseudo-lymphomatous', and were therefore difficult to identify among the normal BM population. In two successive stagings, more than 10% of UJ13A positive isolated mononuclear cells were detected. Some of these cells (1–2%) were HSAN 1-2 positive on one occasion; this patient is clinically stable 3 months after the last staging procedure. The other 3 cases with clearly identified UJ13A positive cells are not clinically evaluable at this time.

A further 9 cases with UJ13A positivity also showed abnormality on both trephines and aspirates. In 8 patients, the immunological studies were in concordance with cyto-histology. For the ninth and as mentioned above, malignant cells clearly observed on aspirates were not identified either on cytological smears or immunological samples, suggesting the loss of malignant clumps on ficoll. Finally, the immunological results were also helpful in patients where the results of trephine biopsies and aspirates differed (11 cases). In 9 cases of this group trephines were classified as abnormal and aspirates normal. Immunological studies confirmed the presence of isolated tumour cells in 5/9 of these cases with further suggestive, but not conclusive, findings in favour of tumour cell involvement (i.e. $\sim$1% cells positive). In 2 cases where aspirates were found abnormal and trephines normal, immunological studies identified the malignant cells only in one case. Finally, in 3/56 cases, the immunological analysis of bone marrow was not possible with UJ13A and HSAN 1-2 antibodies since the initial tumour did not express the corresponding antigens.

### Discussion

The accurate assessment of BM status is one of the important steps in the management of children with neuroblastoma. The metastatic spread may be focal. As a consequence the analysis of trephines and samples taken from several sites may improve diagnosis (Franklin *et al.*, 1983; Bostrom *et al.*, 1985; Bayle *et al.*, 1985). However, no study has attempted to define the number of sites and type of investigation that will maximize tumour cell detection without overloading the laboratory. From our study it appears that biopsies are more helpful at detecting malignant cells than aspirates. In 63.2% of pathological cases, biopsy was the only technique capable of demonstrating tumour, probably because neuroblastoma cells are less easily recognized when the clumps are dissociated. However, both techniques are necessary as in 13% of cases only aspirates were positive. The focal nature of the disorder was demonstrated by the fact that in 36.7% of cases only 1/8 assays (4 biopsies + 4 trephines) were positive, indicating the need to analyse at least 4 sites by both methods. Nevertheless, the analysis of aspirates from additional sites such as sternum and tibia did not yield better detection. Analysis of trephine imprints was also unproductive. Furthermore, Bayle *et al.* (1985) suggested that cytocentrifuged smears of BM mononuclear cells should improve the detection of small aggregates of neuroblastoma cells, mainly by eliminating contaminating red cells. In our limited experience, analysis of these smears did not add useful observation to the diagnosis made on trephines and/or aspirates. Several authors have attempted to improve the detection rate of BM invasion by immunological methods (Reynolds *et al.*, 1982; Kemshead *et al.*, 1983), and our study also demonstrates that immunological analysis improves cytological diagnosis since the immunological findings were pathological in 9 cases of positive biopsies with negative aspirates. In addition, as described for 11 cases in this study, immunological staining may detect neuroblastoma cells which are not yet recognizable by traditional cytological or histological criteria. Such cells need now to be fully characterized in their cytological aspect and membrane marker expression in order to investigate whether the presence of such cells precedes clinical relapse. The value of immunological analysis for the detection of minimal residual BM involvement should then lead to the further development of the procedure. Indeed, the MoAb panel used in this study was too restricted since it did not recognize malignant cells in 5% of cases; the MoAbs were not fully specific for neuroblastoma and for this reason it was difficult to ascertain whether $<$1% isolated positive cells were normal haematopoietic progenitors or neuroblastoma cells. Limitations of the method described here will be solved by using a larger panel of MoAbs, including some which recognize cytoplasmic antigens (Gross *et al.*, 1986) and by characterizing their reactivity on neuroblastoma cells in trephine biopsies, as described by Chilosi *et al.* (1983).

This work was supported by grant no. 6519 from the Association pour la Recherche sur le Cancer (A.R.C.).

### References

ALLAN, P.M., GARSON, J.A., HARPER, E.I., ASSER, U., COAKHAM, H.B., BROWNELL, B. & KEMSHEAD, J.T. (1983). Biological characterization and clinical applications to a monoclonal antibody recognising an antigen restricted to neuroectodermal tissues. *Int. J. Cancer*, **31**, 591.

BAYLE, C., ALLARD, T., RODARY, C., VANDERPLANCKE, J., HARTMAN, O. & LEMERLE, J. (1985). Detection of bone marrow involvement by neuroblastoma: Comparison of two cytological methods. *Eur. Paediatr. Haematol. Oncol.*, **2**, 123.

BOSTROM, B., NESBIT, M.E. Jr. & BRUNNING, R.D. (1985). The value of bone marrow trephine biopsy in the diagnosis of metastatic neuroblastoma. *Amer. J. Ped. Hematol. Oncol.*, **7**, 303.

CHILOSI, M., PIZZOLO, G., FIORE-DONATI, L., BOFILL, M. & JANOSSY, G. (1983). Routine immunofluorescent and histochemical analysis of bone marrow involvement of lymphoma/leukaemia: The use of cryostat sections. *Br. J. Cancer*, **48**, 763.

D'ANGIO, G.J., AUGUST, C., ELKINS, W., EVANS, A.E., SEEGER, R., LENARSKY, C., FEIG, S., WELLS, J., RAMSAY, N., KIM, T., WOODS, W., KRIVIT, W., STRANDJORD, S., COCCIA, P. & NOVAK, L. (1985). Metastatic neuroblastoma managed by supralethal therapy and bone marrow reconstitution (BMRc). Results of a four-institution children's cancer study group pilot study. In *Advances in Neuroblastoma Research,* Evans, A.E. *et al.* (eds) p. 557. A. Liss: New York.

DONNER, L., TRICHE, T.J., ISRAEL, M.A., SEEGER, R.C. & REYNOLDS, C.P. (1985). A panel of monoclonal antibodies which discriminate neuroblastoma from Ewing's sarcoma, rhabdomyosarcoma, neuro-epithelioma and hematopoietic malignancies. In *Advances in Neuroblastoma Research,* Evans, A.E. *et al.* (eds) p. 347. A. Liss: New York.

FAVROT, M.C., PHILIP, I., PHILIP, T., PORTOUKALIAN, J. & DORE, J.F. (1984). Distinct reactivity of Burkitt cell lines with eight monoclonal antibodies correlated with the ethnic origin. *J. Natl Cancer Inst.,* **73,** 841.

FRANKLIN, I.M. & PRITCHARD, J. (1983). Detection of bone-marrow invasion by neuroblastoma is improved by sampling two sites with both aspirates and trephine biopsies. *J. Clin. Pathol.,* **36,** 1215.

GROSS, N., BECK, D., CARREL, S. & MUNOZ, M. (1986). Highly selective recognition in human neuroblastoma cell by mouse monoclonal antibody to a cytoplasmic antigen. *Cancer Res.,* **46,** 2988.

KEMSHEAD, J.T., GOLDMAN, A., FRITSCHY, J., MALPAS, J.S. & PRITCHARD, J. (1983). Use of panels of monoclonal antibodies in the differential diagnosis of neuroblastoma and lymphoblastic disorders. *Lancer,* **i,** 12.

PHILIP, T., ZUCKER, J.M., FAVROT, M., BORDIGONI, P., PLOUVIER, E., ROBERT, A., BERNARD, J.L., SOUILLET, G., PHILIP, I., LUTZ, J.P., CARTON, P. & KEMSHEAD, J.T. (1985*a*). Purged autologous bone marrow transplantation in 25 cases of very poor prognosis neuroblastoma. *Lancet,* **ii,** 576.

PHILIP, T., BERNARD, J.L., ZUCKER, J.A. & 9 others. (1985*b*). Massive consolidation therapy followed by bone marrow transplantation in neuroblastoma: An unselected group of stage IV over one year of age. *J. Clin. Oncol.,* (in press).

REYNOLDS, C.P. & GRAHAM SMITH, R. (1982). A sensitive immunoassay for human neuroblastoma cells. In *Hybridomas in cancer diagnosis and treatment,* Mitchell, N.S. & Oettgen, H.F. (eds) p. 235. Raven Press: New York.

SHAFFORD, E.A., ROGERS, D.W. & PRITCHARD, J. (1984). Advanced neuroblastoma: Improved response rate using a multiagent regimen (OPEC) including sequential cisplatin and VM-26. *J. Clin. Oncol.,* **2,** 742.